# Evaluating the association of social needs assessment data with cardiometabolic health status in a federally qualified community health center patient population

**DOI:** 10.1186/s12872-021-02149-5

**Published:** 2021-07-14

**Authors:** Connor Drake, Tyler Lian, Justin G. Trogdon, David Edelman, Howard Eisenson, Morris Weinberger, Kristin Reiter, Christopher M. Shea

**Affiliations:** 1 Department of Population Health Sciences, Duke University School of Medicine, 215 Morris Street, Durham, NC 27701 USA; 2 Department of Health Policy and Management, Gillings School of Global Public Health, University of North Carolina at Chapel Hill, 135 Dauer Dr, Chapel Hill, NC 27519 USA; 3 Department of Medicine, Duke University School of Medicine, 2301 Erwin Rd, Durham, NC 27705 USA; 4 Durham VA Healthcare System, 508 Fulton St, Durham, NC 27705 USA; 5 Lincoln Community Health Center, 1301 Fayetteville St, Durham, NC 27707 USA; 6 Department of Family Medicine and Community Health, Duke University School of Medicine, DUMC 2914, Durham, NC 27710 USA

**Keywords:** Social determinants of health, Social needs, Primary care, Predictive analytics, Electronic health record

## Abstract

**Background:**

Health systems are increasingly using standardized social needs screening and response protocols including the Protocol for Responding to and Assessing Patients’ Risks, Assets, and Experiences (PRAPARE) to improve population health and equity; despite established relationships between the social determinants of health and health outcomes, little is known about the associations between standardized social needs assessment information and patients’ clinical condition.

**Methods:**

In this cross-sectional study, we examined the relationship between social needs screening assessment data and measures of cardiometabolic clinical health from electronic health records data using two modelling approaches: a backward stepwise logistic regression and a least absolute selection and shrinkage operation (LASSO) logistic regression. Primary outcomes were dichotomized cardiometabolic measures related to obesity, hypertension, and atherosclerotic cardiovascular disease (ASCVD) 10-year risk. Nested models were built to evaluate the utility of social needs assessment data from PRAPARE for risk prediction, stratification, and population health management.

**Results:**

Social needs related to lack of housing, unemployment, stress, access to medicine or health care, and inability to afford phone service were consistently associated with cardiometabolic risk across models. Model fit, as measured by the c-statistic, was poor for predicting obesity (logistic = 0.586; LASSO = 0.587), moderate for stage 1 hypertension (logistic = 0.703; LASSO = 0.688), and high for borderline ASCVD risk (logistic = 0.954; LASSO = 0.950).

**Conclusions:**

Associations between social needs assessment data and clinical outcomes vary by cardiometabolic condition. Social needs assessment data may be useful for prospectively identifying patients at heightened cardiometabolic risk; however, there are limits to the utility of social needs data for improving predictive performance.

**Supplementary Information:**

The online version contains supplementary material available at 10.1186/s12872-021-02149-5.

## Background

Social determinants of health (SDOH), such as access to nutritious food, transportation, employment, and stable housing, are significant drivers of health outcomes [1, 2]. For health care systems to successfully reform delivery towards value, prevention, and effective population health management, they need to assess and respond to social needs associated with downstream consequences of the SDOH [3, 4]. To this end, health care systems and payers are increasingly collecting population- and individual-level data on social needs, including food insecurity, unemployment, housing instability, and transportation barriers [5, 6].

In 2016, the Protocol for Responding to and Assessing Patients’ Assets, Risks, and Experiences (PRAPARE) was developed by the National Association of Community Health Centers and partnering organizations as a screening tool and corresponding clinical workflow to assess and respond to patients’ social needs. PRAPARE has been the most prevalent social needs assessment in the United States, increasingly used by hospitals, health systems, and health plans [7, 8]. The PRAPARE screening assessment bridges social risk and clinical risk indicators by being embedded into electronic health record (EHR) systems and has facilitated national standards surrounding social risk data capture, reporting, and population health and care management activities [9].

Despite these features and the prevalence of PRAPARE, there is little evidence on the relationship between data from PRAPARE and clinical outcomes of interest [10]. A recent systematic review of PRAPARE and similar social needs screening assessments found little evidence to evaluate predictive validity [11]. Addressing this gap is critical as health care systems consider delivery reforms that embrace population health management and care coordination across the health and social care continuum [4, 12]. With a better understanding of how social needs screening assessment data predicts clinical risk, health systems and payers can identify patients with health related social needs that are most predictive of poor health, provide care management to promote linkages to appropriate wraparound services and community resources to patients most likely to realize health benefits, measure the impact of interventions, manage patient panels, and inform care team composition [13]. As health systems and payers increasingly invest in collecting this information [4, 14, 15], there is a need to evaluate the relationship between patients’ social needs and their clinical risk to design tailored interventions.

This cross-sectional study examined the relationship between responses to PRAPARE and cardiometabolic clinical outcomes among patients in a federally-qualified community health center (FQHC). We utilized two approaches to examine the association between social needs assessment data and the likelihood of the following clinical indicators of cardiometabolic health status: obesity, hypertension, and atherosclerotic cardiovascular disease (ASCVD) 10-year risk. These outcomes are important because cardiometabolic disease is the leading cause of death of people in the United States, and obesity and hypertension are both modifiable risk factors [16]. Our goals were to (1) better understand the social needs and health status of a defined population and (2) evaluate PRAPARE social needs assessment data’s association with cardiometabolic health status to inform risk prediction, stratification, and population health management. We hypothesized that models using social needs data from PRAPARE would have moderate performance for three cardiometabolic health outcomes.

## Methods

### Study setting and data collection

The study was conducted at a FQHC in a medium-sized city in the southeastern United States. In 2019, the FQHC saw 36,361 unique patients, 97% of whom had incomes ≤ 200% of the federal poverty level; 57% were uninsured and 93% were members of racial and/or ethnic minorities.

The FQHC began implementing PRAPARE in mid-2017 in its Pediatric, Adult Medicine, and Family Medicine clinics. PRAPARE tool was fully integrated within the FQHC’s EHR. The social needs assessment is administered via patient interview, and referrals to community resources or social services are made based on identified needs. Additional detail on the FQHC’s clinical workflow, patient population, EHR integration, and implementation logistics are published elsewhere [17].

### Measures

We obtained the data used in this analysis through a retrospective query to abstract charts of patients who had received PRAPARE as part of their primary care clinical encounter. PRAPARE includes a set of national, well-validated core measures and additional optional measures to match community priorities [18]. The core measures evaluate the following: race, ethnicity, education, employment, migrant/seasonal farm work, insurance, veteran status, income, language, material security (food, clothing, childcare, utilities, medicine/health care, phone, and other), transportation, housing status, housing stability, social integration and support, address/neighborhood, and stress. The optional measures include incarceration history, safety, refugee status, and domestic or interpersonal violence. PRAPARE aligns with existing national initiatives [19], ICD-10 clinical coding, and the Uniform Data System used by the Health Resources and Services Administration. All core and optional measures were included as independent variables except for neighborhood, incarceration history, refugee status (not consistently collected during the chart abstraction period), language (data missingness and correlation with ethnicity), and income (data quality/missingness). Sex and age were included as covariates in the analysis.

We evaluated three cardiometabolic clinical outcomes: body mass index (BMI), systolic and diastolic blood pressure, and ASCVD 10-year risk. These outcomes were selected because of their relevance in primary care [20], data availability and integrity, and causal links to how social needs can affect clinical risk [21]. All three clinical outcomes were dichotomized: obesity was defined as BMI ≥ 30 for all patients with recorded height and weight (n = 2153). Hypertension was defined by a stage-1 threshold using patients’ systolic (> 130 mmHg) or diastolic (> 80 mmHg) blood pressure (n = 2174) [22]. Finally, the ASCVD risk score is an estimate of the likelihood of an ASCVD event over the following 10 years and was developed to identify patients that might benefit from primary prevention [23]. Patients with a risk score > 7.5% were classified as being at risk requiring clinical intervention using statin therapy based on existing guidelines [24]. Because ASCVD is not a valid cardiovascular estimate for patients younger than 40 years, they were excluded, leading to an analytic sample of 1468 patients after imputing missing cholesterol readings with a healthy value as is consistent with best practices for managing missing EHR data [25].

### Statistical analysis

#### Regression methods

We employed two standard model selection approaches to assess the associations between PRAPARE responses and the three clinical outcomes: (1) backwards stepwise logistic regression, a parametric approach in which predictor variables are included in the model and removed individually if they were not statistically significant at a 0.157 level, a value recognized to optimize the Akaike information criteria (AIC) [26, 27]; and (2) logistic least absolute selection and shrinkage operation (LASSO) regression, a type of supervised machine learning algorithm that performs model selection by “shrinking” or penalizing variables, (i.e., setting certain coefficients to zero if they are not contributing explanatory power to the model) [28]. As a result, LASSO is designed to avoid overfitting better than regression models without a penalization function and uses a data-driven approach to select variables, minimize prediction error, maximize out-of-sample performance, and address issues with multicollinearity. We used three different LASSO models with different model selection criteria. One was based on an adaptive LASSO, one minimized the Bayesian information criteria (BIC), and one minimized AIC, which estimates the amount of information lost by using the model [29]. Model fit was similar across all three LASSO approaches, but the AIC approach consistently performed well and was selected based on performance and theory. Additional results comparing the different LASSO approaches for all variables of interest can be found in Additional file 1.

For each outcome and regression approach, nested sequential models were built by adding social need and demographic covariates in groupings. This sequential approach highlighted changes in model performance and goodness-of-fit as PRAPARE covariates are added to the limited set of demographic characteristics usually operationalized in typical EHR. Model 1 used only age and sex; Model 2 added race and ethnicity; and Model 3 added expanded demographic covariates from the PRAPARE that can often be found in existing EHR data (e.g., household size, education, employment, insurance status). The full model, Model 4, added the remaining social needs covariates identified during screening. For each model, the backwards stepwise or LASSO approach selected variables to include. Excluded variables were also excluded in all subsequent models to enable direct comparison of the nested models.

#### Model evaluation

To evaluate model goodness-of-fit, a likelihood-ratio test was conducted for each successive pair of nested models. To evaluate model performance, we computed the concordance statistic (c) and assessed the statistical significance of c-statistic improvement between sequential models using a equality-of-areas test [30]. The c-statistic represents the area under the receiver operating characteristic curve, which can range from 0 to 1. A c-statistic of 0.5 indicates that the model performs as well as random chance at classifying outcomes and 1 indicates perfect accuracy. For the stepwise logistic regression models, c-statistics were computed over the full dataset; for the LASSO regression models, c-statistics were computed over a validation dataset that contained 20% of the observations, which were excluded from model training. We hypothesized that model performance for the full PRAPARE model (Model 4) would be satisfactory across the three clinical outcomes. We defined satisfactory performance as having a c-statistic > 0.65, the lower bound for moderate discrimination [31]. All statistical analysis was conducted in Stata version 16 [32].

## Results

### Study sample

Between May 2017 and February 2019, PRAPARE was delivered to 2192 patients, primarily those who were referred to behavioral health either as part of a primary care or stand-alone appointment (Table 1). The average patient age was 50 and ~ 60% of patients were female. Almost half of patients were African American and about a third were Hispanic. Approximately one third of patients lacked a high school education and the majority were uninsured or unemployed. The most commonly-reported social needs were social isolation, barriers to health care and medicine access, lack of housing, transportation barriers, food insecurity, and high stress. Over half of patients (54.5%) were obese, 52.8% had stage-1 hypertension, and 73.6% had borderline 10-year ASCVD risk or higher. Since the calculation of ASCVD risk is only valid for patients age 40–79, the analytic subsample for the ASCVD model is smaller (n = 1468); including a smaller proportion of Hispanic/Latino patients (24.4% vs. 35.1% overall).

**Table 1:** Description of differences between patients across clinical risk indicators

	Overall	Obese			High BP, Stage 1			ASCVD Risk		
		−	+	*P* value	−	+	*P* value	−	+	*P* value
N	2192	980	1173	–	1027	1147	–	387	1081	–
*Demographics*^a^										
Age, year, mean	49.6	49.2	50.2	0.102	47.9	54.8	< 0.001*	47.4	58.7	< 0.001*
Female	61.8	53.3	68.8	< 0.001*	63.9	55.6	< 0.001*	79.1	52.5	< 0.001*
Race										
Black/African American	49.5	45.5	52.6	0.001*	43.1	66.5	< 0.001*	26.1	71.3	< 0.001*
White/Caucasian	14.0	15.8	12.4	0.021*	14.9	11.6	0.050*	18.1	14.1	0.058
Other	26.7	28.5	25.5	0.120	30.5	16.5	< 0.001*	55.8	14.6	< 0.001*
Not reported/declined	9.9	10.2	9.6	0.611	11.5	5.4	< 0.001*	–	–	–
Hispanic/Latino	35.1	36.4	34.4	0.330	40.7	19.9	< 0.001*	56.8	12.8	< 0.001*
Members per household										
Lives alone	27.9	30.1	26.2	0.059	25.8	33.7	0.001*	16.3	40.5	< 0.001*
Two	22.8	22.0	23.5	0.450	21.3	27.0	0.008*	18.3	26.5	0.002*
Three to four	30.9	30.8	31.0	0.914	32.6	26.0	0.005*	40.3	22.9	< 0.001*
More than five	18.4	17.1	19.3	0.215	20.3	13.3	< 0.001*	25.1	10.1	< 0.001*
Migrant or seasonal work	0.8	0.9	0.7	0.753	0.7	0.9	0.647	0.8	0.5	0.479
Military discharge	1.6	1.8	1.4	0.405	1.7	1.3	0.576	0.3	2.9	0.004*
Uninsured	58.4	60.5	56.7	0.081	59.7	55.4	0.081	68.3	43.6	< 0.001*
Lacks high school education	34.6	35.3	33.8	0.502	35.8	31.8	0.099	41.8	29.5	< 0.001*
Work situation										
Full-time	24.8	25.0	25.1	0.959	27.4	18.0	< 0.001*	34.3	15.5	< 0.001*
Part-time	19.6	20.9	18.5	0.168	21.3	14.6	0.001*	26.8	13.5	< 0.001*
Unemployed, seeking work	26.6	25.8	27.5	0.409	24.3	33.5	< 0.001*	23.2	31.7	0.002*
Unemployed, not seeking work	29.0	28.3	29.0	0.726	27.1	33.9	0.003*	15.8	39.3	< 0.001*
*Social needs*^a^										
No housing	19.4	22.3	16.6	0.001*	18.7	21.4	0.167	15.3	21.6	0.009*
Worried about losing housing	14.3	14.7	14.1	0.721	14.4	14.1	0.859	20.2	12.1	< 0.001*
Lacks transportation	17.6	19.7	15.7	0.022*	17.3	18.2	0.656	17.7	17.4	0.890
Low social interaction	37.0	37.3	36.4	0.658	36.3	38.6	0.339	34.4	37.9	0.237
High stress	14.3	16.3	12.2	0.009*	13.8	15.4	0.354	17.8	13.0	0.024*
Feels unsafe at residence	7.7	8.1	7.3	0.548	7.8	7.6	0.862	10.1	8.0	0.210
Afraid of partner	3.7	3.4	3.7	0.675	4.1	2.6	0.138	3.6	2.2	0.149
Other material need										
Food	16.2	15.9	16.4	0.738	14.9	20.0	0.006*	10.6	20.4	< 0.001*
Access to health care	19.1	20.2	18.0	0.207	17.1	25.4	< 0.001*	16.3	21.6	0.027*
Utilities	7.3	7.4	0.7.2	0.840	6.7	8.9	0.097	6.5	8.0	0.355
Clothing	4.4	5.3	3.6	0.074	4.0	5.6	0.136	3.0	5.1	0.098
Child care	1.5	1.4	1.5	0.783	1.5	1.3	0.714	0.3	0.7	0.370
Phone	2.6	2.7	2.5	0.842	1.8	4.8	< 0.001*	1.1	3.4	0.020*
Other	7.0	7.5	6.4	0.345	7.3	5.9	0.275	6.8	6.9	0.925

^a^Reported as %, unless otherwise specified

*P < 0.05

The presence of social needs was generally greater among patients with adverse health outcomes (Table 1). In bivariate analyses, food insecurity, lack of access to care and medicine, and inability to afford a phone plan were significantly more prevalent in patients with high blood pressure and borderline-or-higher ASCVD risk than in patients without. However, this trend was not the case for obese patients, who were more likely to have housing, had fewer transportation barriers, and lower stress.

### Nested models

For both the stepwise logistic and LASSO approaches, the full models (Model 4) for all three clinical outcomes included both demographic and social need variables (Additional file 2). The number of variables retained in each model ranged between 7 and 13 for the stepwise logistic regression models and between 5 and 13 for the LASSO logistic regression models. The variables that were included in at least three models were: age, sex, race, lack of housing, unemployment, high stress, and access to health care or medicine.

In multivariable analyses, the magnitude and direction of the odds ratios were similar between the stepwise and LASSO approaches (Table 2). High stress and lack of housing were associated with decreased odds of obesity in full models. Identified needs related to health care and medicine access were associated with increased odds of both hypertension and borderline-or-higher ASCVD risk. Lack of housing was associated with lower odds of obesity, but higher odds of borderline-or-higher ASCVD risk.

**Table 2:** Estimated coefficients for final models (Model 4s)

	Obesity		Hypertension, stage-1		ASCVD, borderline	
	Stepwise^a^	LASSO^b^	Stepwise^a^	LASSO^b^	Stepwise^a^	LASSO^b^
Age	1.01 (1.00, 1.01)	1.01	1.04 (1.03, 1.05)	1.04	1.27 (1.22, 1.31)	1.26
Female	2.08 (1.71, 2.54)	2.13	0.76 (0.62, 0.93)	0.77	0.10 (0.06, 0.16)	0.10
Race						
Ref: Black/African Am			Ref	Ref	Ref	Ref
White/Caucasian			0.50 (0.36, 0.68)	0.44	0.14 (0.08, 0.25)	0.18
Other			0.41 (0.32, 0.52)	0.42	0.13 (0.06, 0.31)	0.16
Not reported/declined			0.42 (0.30, 0.59)	0.46		
Hispanic/Latino				0.88	0.57 (0.25, 1.28)	0.60
Members per household						
Ref: One			Ref	Ref		
Two			1.25 (0.98, 1.60)	1.19		
Migrant or seasonal work			3.45 (1.01, 11.8)	3.99		
Military discharge				1.98		
Uninsured				1.23	1.18 (0.75, 1.86)	
Work situation						
Ref: Full-time	Ref		Ref	Ref	Ref	Ref
Part-time	0.79 (0.62, 1.02)					
Unemp., seeking work					1.77 (1.10, 2.85)	
Unemp., not seeking work	0.85 (0.67, 1.08)		0.79 (0.62, 1.00)	0.79	1.63 (0.93, 2.85)	1.73
No housing	0.75 (0.59, 0.95)	0.86				1.09
Lacks transportation	0.80 (0.62, 1.03)	0.83				
Low social interaction						1.08
High stress	0.66 (0.50, 0.88)	0.74			0.63 (0.26, 1.11)	0.76
Feels unsafe at residence					0.56 (0.28, 1.13)	
Other material need						
Food					1.80 (0.96, 3.36)	1.32
Access to health care			1.53 (1.19, 1.97)	1.16	1.67 (0.97, 2.87)	1.17
Child care						2.71
Phone					3.65 (0.78, 17.0)	2.27

^a^Odds ratios and 95% confidence intervals (lower, upper) reported

^b^Odds ratios reported; the LASSO algorithm does not compute *P* values

For all three clinical outcomes, the final stepwise and LASSO models performed similarly, as measured by c-statistic (Table 3). Model performance was poor for predicting obesity (stepwise, c = 0.617; LASSO, c = 0.590), moderate for hypertension (stepwise, c = 0.711; LASSO, c = 0.681), and high for ASCVD risk (stepwise, c = 0.944; LASSO, c = 0.949). The high prediction performance for ASCVD risk was expected as age, race, and sex are used to calculate the score and were included as covariates alongside the PRAPARE variables.

**Table 3:** Performance and goodness-of-fit of nested models

Outcome	Model	C-statistic	95% CI, LL	95% CI, UL	Comparison of C-index (*P* value)^a^	Likelihood-ratio test (*P* value)^a^
Backwards stepwise logistic						
Obesity	M1	0.5941	0.5675	0.6208	–	–
	M2	0.5941	0.5675	0.6208	1.0000	1.0000
	M3	0.5992	0.5728	0.6257	0.4163	0.0836
	M4	0.6173	0.5912	0.6433	0.0133*	0.0002*
Hypertension, stage-1	M1	0.6670	0.6421	0.6920	–	–
	M2	0.7013	0.6771	0.7256	< 0.0001*	< 0.0001*
	M3	0.7059	0.6818	0.7299	0.0991	0.0106*
	M4	0.7111	0.6872	0.7350	0.0531	0.0009*
ASCVD, borderline	M1	0.9021	0.8852	0.9191	–	–
	M2	0.9368	0.9228	0.9508	< 0.0001*	< 0.0001*
	M3	0.9389	0.9253	0.9526	0.1197	0.0116*
	M4	0.9436	0.9307	0.9565	0.0087*	0.0011*
LASSO						
Obesity	M1	0.5654	0.5052	0.6256	–	–
	M2	0.5654	0.5052	0.6256	1.0000	1.0000
	M3	0.5654	0.5052	0.6256	1.0000	1.0000
	M4	0.5896	0.5305	0.6487	0.1301	0.0038*
Hypertension, stage-1	M1	0.6358	0.5778	0.6938	–	–
	M2	0.6749	0.6189	0.7308	0.0230*	< 0.0001*
	M3	0.6770	0.6212	0.7329	0.6437	0.0546
	M4	0.6806	0.6250	0.7362	0.3969	0.0050*
ASCVD, borderline	M1	0.8880	0.8458	0.9303	–	–
	M2	0.9505	0.9259	0.9751	< 0.0001*	< 0.0001*
	M3	0.9486	0.9234	0.9738	0.1339	0.0583
	M4	0.9488	0.9241	0.9736	0.9281	0.0170*

^a^Compared with previous model

*P < 0.05

With each sequential model, improvements in performance were observed as covariates were added; however, improvements were not always statistically significant (Table 3). For obesity, the addition of the social needs covariates (Model 4) resulted in a statistically significant increase in the c-statistic, but only in the stepwise approach. For hypertension, only the inclusion of race and ethnicity covariates (for Model 2) was associated with a significant increase in the c-statistic for both approaches. For ASCVD risk, the addition of the social needs covariates (for Model 4) significantly increased the c-statistic for the stepwise approach; however, for the LASSO approach, the c-statistic plateaued in Models 2, 3 and 4, with a slight decrease in performance in Models 3 and 4.

Sequential models often demonstrated statistically significant improvements in goodness-of-fit (Table 3). In particular, for both regression approaches, the addition of the social needs covariates from PRAPARE in all three clinical outcomes significantly improved goodness-of-fit compared to the model that included extended demographics only.

## Discussion

Assessing and responding to social needs is a major priority for health care systems seeking to deliver high-value care and improve population health. Efforts to better integrate these activities into routine clinical encounters and standard of care [6] include social needs EHR documentation strategies [33], innovative care models [34, 35], and cross-sector collaboration [36]. This study builds on the existing literature by evaluating the relationship between responses to a standardized social needs assessment and accepted measures of cardiometabolic health outcomes. Our intention is to highlight practical analytical tools for leveraging social needs information from PRAPARE and similar screening tools [37, 38] to better understand the association between social needs and commonly-studied cardiometabolic outcomes. The application of these analytical tools has the potential to enhance value-based care, population health management, panel management [13], and integrated social-medical care model design and implementation [39, 40].

We evaluated the relationship between data from PRAPARE and three cardiometabolic outcomes using two predictive analytic approaches. We found that social needs were more prevalent in patients with hypertension and borderline-or-higher ASCVD risk. Interestingly, social needs were less prevalent in obese patients compared to those who were not obese. Lack of housing, high stress, and access to medicine and health care were the only social needs that were selected in models across more than one clinical risk outcome. These social needs may be proxies for additional, interrelated non-medical drivers of health and do not represent causal mechanisms between social needs and clinical outcomes.

The presence of social needs was associated with lower prevalence of obesity. Existing literature indicates that this counter-intuitive finding may be because obesity has a unique and multifactorial relationship to social needs and SDOH that varies by cultural context, race, ethnicity, and gender [41]. This finding highlights the importance of both understanding that associations between social needs and clinical outcomes depend on how adverse outcomes are defined, as well as the need for cautious interpretation of the directionality of the effect based on the variables retained in the model.

We hypothesized that the predictive analytic approaches would demonstrate moderate performance for all three cardiometabolic outcomes (c-statistic > 0.65); we found support for this hypothesis for ASCVD risk and hypertension, but not for obesity. We noted that model performance for ASCVD risk was very high even without including the clinical parameters or health behaviors used to calculate an ASCVD risk score as predictor variables (blood pressure, total and high density lipoprotein cholesterol, diabetes diagnosis, smoking status, and hypertension treatment). The comparison across nested models further contextualizes this finding. The inclusion of social needs variables for Model 4 resulted in a statistically significant increase in prediction performance compared to Model 3 in the backwards stepwise approach. The small decrease in the LASSO approach performance between the same models may be due to overfitting on a small sample, despite regularization to prevent it, and limitations to increases to prediction performance with increasingly granular data on an individual’s unmet social needs. Nevertheless, this still suggests that social needs assessment data as a whole has useful associations with clinical outcomes in the absence of information on health behaviors and biometric data.

We found that the stepwise logistic and machine learning LASSO regression models demonstrated similar performance, a finding consistent with prior studies assessing the performance of predictive models [42, 43]. A potential explanation is that the advantages of more advanced, resource-intensive machine learning techniques like LASSO regression or random forest models, compared to stepwise logistic regression models, require more observations and dimensionality to become apparent [31]. The number and functional form of included variables also can influence results, with similar research demonstrating better performance for machine learning approaches when more variables and continuous variables are used [44]. This underscores the importance of considerations regarding data transformation, variable functional form, and data missingness when using and selecting a predictive analytical approach.

This study has several limitations. Because PRAPARE was administered only to a subset of patients referred to behavioral health, there was less variation in social need levels to base predictive analytics. The smaller sample size of patients from multiple clinics within a FQHC may have limited prediction performance and generalizability. Generalizability may also be limited by the setting—FQHCs in the southeastern United States. Finally, the prediction modeling approaches used in this analysis do not allow for making conclusions on potential causal inference. Despite these limitations, this study contributes to an emerging evidence base that suggests the formal and pragmatic validity of PRAPARE and provides insights into how social needs data could be used in outpatient settings to predict cardiometabolic health outcomes.

Predictive analytics may have the potential to proactively identify patients at higher risk for poor health outcomes who could benefit from an intervention, even in the absence of data obtained in current screening methods for cardiometabolic outcomes; our results align with previous literature suggesting limits to the utility of SDOH data for risk prediction [45]. In other words, additional SDOH data do not always lead to statistically significant improvements in prediction performance. This is relevant as payors, including state Medicaid programs [46], collect social needs data for new enrollees to identify patients at risk for worsening medical complexity based on social needs in order to improve population health. Our findings suggest that performance may depend on how clinical outcomes are defined and that relationships between social needs assessment data and outcomes vary by disease pathway.

### Future research directions

Future research should evaluate social needs prevalence and association with additional clinical outcomes, using prospective data to understand how social needs data can be used to predict clinical risk and, ultimately, improve population health. Though this study focused on moderate clinical outcomes that may be useful for proactive intervention, outcomes that correspond to more severe clinical conditions (e.g., A1c > 8.0%, ASCVD > 20%) may have differing and perhaps stronger associations with unmet social needs. Ideally, this would include linking multiple data sources to comprehensively describe patient behaviors and environment in addition to information on social needs to predict other clinical risk and health status indicators including uncontrolled diabetes, co-morbidity burden, and behavioral health outcomes. Moreover, risk prediction around social needs will only add significant value if it is coupled with implementing evidence based responses to social needs that meaningfully address social needs to improve health outcomes in a cost-effective manner. These responses, or social care interventions, will need to be rigorously tested in diverse settings among a study sample of sufficient size to detect its impact on outcomes of interest including medication adherence, utilization, and cardiovascular health status.

Understanding the relationship between clinical outcomes and social needs may have important ramifications for how payers adjust for risk. In addition, future research should also evaluate the relationship between social needs assessment data and the likelihood of requiring costly types of heath care utilization including inpatient and emergency department visits. As social need screening becomes wider spread, there is a need to understand how this data can be used to improve health equity as health systems focus on improving population health. For example, our findings and future research can inform the business case for health systems to implement interventions to address social needs, which has the potential to narrow disparities in care resulting from social and economic inequities [47]. A critical step will be to design quality measures that complement care guidelines to focus support on medically vulnerable patients with unmet social needs [48].

## Conclusions

Associations between standardized social needs assessment data and clinical outcomes vary by cardiometabolic outcome and may depend on how clinical outcomes are defined, which has implications for designing population health management and quality improvement initiatives using social needs assessment data on a defined patient population. Predictive analytics has the potential to leverage associations between clinical outcomes and social needs assessment data; however, there are limits to the utility of social needs data for improving predictive performance. Future research should further evaluate the utility of social needs assessment data to predict forms of clinical risk and health care utilization, especially as this data becomes more available in administrative or health records.

## Supplementary Information

**Additional file 1**. Comparison across LASSO logistic regressions across the three clinical outcomes. Models and c-statistics for cross-validation, minimum-AIC, minimum-BIC, and adaptive LASSO models.

**Additional file 2**. Included covariates for logistic nested models. Description of four nested models for stepwise logistic and LASSO approaches.

## Data Availability

The data that support the findings of this study are available upon reasonable request from the corresponding author CD. The data are not publicly available due to them containing information that could compromise research participant privacy.

## Abbreviations

AIC: Akaike information criterion
ASCVD: Atherosclerotic cardiovascular disease
BIC: Bayesian information criterion
BMI: Body mass index
EHR: Electronic health record
FQHC: Federally qualified health center
LASSO: Least absolute shrinkage and selection operator
OR: Odds ratio
PRAPARE: Protocol for responding to and assessing patients’ assets, risks, and experiences
SDOH: Social determinants of health
