# Supplementary material for: Evaluating the association of social needs assessment data with cardiometabolic health status in a federally qualified community health center patient population
Source: BMC Cardiovasc Disord. 2021 Jul 14;21:342. doi: 10.1186/s12872-021-02149-5 (PMC8278633; doi:10.1186/s12872-021-02149-5)
Supplement: Supplementary file 1 — Additional file 1. Comparison across LASSO logistic regressions across the three clinical outcomes. Models and c-statistics for cross-validation, minimum-AIC, minimum-BIC, and adaptive LASSO models. [file 12872_2021_2149_MOESM1_ESM.docx]

Additional material for “Evaluating the Association of Social Needs Assessment Data with Cardiometabolic Health Status in a Federally Qualified Community Health Center Patient Population: A Cross-Sectional Study”

**Table 1. Comparison across LASSO logistic regressions for obesity**

|  | **Obese** | | | |
| --- | --- | --- | --- | --- |
|  | **Cross Valid.** | **minAIC** | **minBIC** | **Adaptive** |
| Age | 1.00 | 1.00 |  | 1.01 |
| Female | 1.85 | 1.85 | 1.51 | 2.13 |
| Race (base = White/Caucasian) |  |  |  |  |
| Black/African American | 1.01 | 1.01 |  |  |
| Asian | 0.46 | 0.46 |  | 0.25 |
| No housing | 0.88 | 0.88 |  | 0.81 |
| Lacks transportation | 0.89 | 0.89 |  | 0.80 |
| High stress | 0.79 | 0.79 |  | 0.67 |
| Constant | 0.76 | 0.76 | 0.87 | 0.63 |
| ***C-statistic*** | 0.5849  (0.5274 – 0.6424) | 0.5849  (0.5274 – 0.6424) | 0.5487  (0.5000 – 0.5975) | 0.5861  (0.5287 – 0.6434) |
| ***Χ^2^ test for C-statistic equality*** | p-value = 0.1623 | | | |

**Table 2. Comparison across LASSO logistic regressions for stage-2 hypertension**

|  | **High BP, Stage 2** | | | |
| --- | --- | --- | --- | --- |
|  | **Cross Valid.** | **minAIC** | **minBIC** | **Adaptive** |
| Age | 1.03 | 1.03 | 1.02 | 1.04 |
| Female | 0.80 | 0.80 | 0.92 |  |
| Race (base = White/Caucasian) |  |  |  |  |
| Black/African American | 1.74 | 1.74 | 1.59 | 2.03 |
| Multiracial | 0.91 | 0.91 |  |  |
| Native Hawaiian/other Pacific Island | 1.91 | 1.91 |  |  |
| Hispanic/Latino | 0.93 | 0.93 | 0.97 |  |
| Military discharge | 0.56 | 0.56 |  | 0.49 |
| No housing | 1.09 | 1.09 |  |  |
| Work situation (base = Full-time) |  |  |  |  |
| Unemployed, seeking work | 1.33 | 1.33 | 1.19 | 1.40 |
| Unemployed, not seeking work |  |  |  |  |
| Uninsured | 1.19 | 1.19 |  | 1.30 |
| Low social interaction | 1.06 | 1.06 |  |  |
| Other self-reported need |  |  |  |  |
| Access to medicine or health care | 1.26 | 1.26 | 1.16 | 1.30 |
| Phone | 2.19 | 2.19 | 1.73 | 2.54 |
| Constant | 0.04 | 0.04 | 0.07 | 0.03 |
| ***C-statistic*** | 0.6842  (0.6280 – 0.7404) | 0.6842  (0.6280 – 0.7404) | 0.6878  (0.6315 – 0.7441) | 0.6893  (0.6330 – 0.7457) |
| ***Χ^2^ test for C-statistic equality*** | p-value = 0.0200 | | | |

**Table 3. Comparison across LASSO logistic regressions for ASCVD risk**

|  | **ASCVD, Borderline** | | | |
| --- | --- | --- | --- | --- |
|  | **Cross Valid.** | **minAIC** | **minBIC** | **Adaptive** |
| Age | 1.23 | 1.25 | 1.19 | 1.27 |
| Female | 0.15 | 0.13 | 0.25 | 0.10 |
| Race (base = White/Caucasian) |  |  |  |  |
| Black/African American | 4.33 | 4.64 | 3.33 | 5.20 |
| Asian | 0.72 | 0.60 |  | 0.49 |
| American Indian/Alaskan Native | 46.99 | 100.3 | 2.18 | 2371.01 |
| Other | 0.63 | 0.60 | 0.75 | 0.55 |
| Hispanic/Latino | 0.71 | 0.71 | 0.72 | 0.68 |
| Military discharge |  | 1.44 |  |  |
| No housing | 1.14 | 1.19 |  | 1.27 |
| Work situation (base = Full-time) |  |  |  |  |
| Unemployed, seeking work | 1.14 | 1.24 |  | 1.40 |
| Unemployed, not seeking work | 1.42 | 1.56 | 1.07 | 1.80 |
| Low social interaction | 1.13 | 1.21 |  | 1.32 |
| High stress | 0.71 | 0.63 |  | 0.54 |
| Feels unsafe at residence | 0.92 | 0.86 |  | 0.80 |
| Other self-reported need |  |  |  |  |
| Food | 1.47 | 1.54 | 1.24 | 1.63 |
| Access to medicine or health care | 1.19 | 1.27 |  | 1.37 |
| Child care | 2.52 | 3.70 |  | 17.39 |
| Phone | 2.37 | 2.70 | 1.40 | 3.54 |
| Other |  | 1.12 |  |  |
| Constant | 0.00 | 0.00 | 0.00 | 0.00 |
| ***C-statistic*** | 0.9495  (0.9262 – 0.9729) | 0.9489  (0.9254 – 0.9725) | 0.9490  (0.9251 – 0.9730) | 0.9475  (0.9236 – 0.9715) |
| ***Χ^2^ test for C-statistic equality*** | p-value = 0.4135 | | | |
