# Supplementary material for: Evaluating the association of social needs assessment data with cardiometabolic health status in a federally qualified community health center patient population
Source: BMC Cardiovasc Disord. 2021 Jul 14;21:342. doi: 10.1186/s12872-021-02149-5 (PMC8278633; doi:10.1186/s12872-021-02149-5)
Supplement: Supplementary file 2 — Additional file 2. Included covariates for logistic nested models. Description of four nested models for stepwise logistic and LASSO approaches. [file 12872_2021_2149_MOESM2_ESM.docx]

Additional material for “Evaluating the Association of Social Needs Assessment Data with Cardiometabolic Health Status in a Federally Qualified Community Health Center Patient Population: A Cross-Sectional Study”

**TABLE 1. Included Covariates for Stepwise Logistic Nested Models**

|  | Obesity | | | | Hypertension, Stage-1 | | | | ASCVD, Borderline | | | |
| --- | --- | --- | --- | --- | --- | --- | --- | --- | --- | --- | --- | --- |
|  | **M1** | **M2** | **M3** | **M4** | **M1** | **M2** | **M3** | **M4** | **M1** | **M2** | **M3** | **M4** |
| Age | ○ | ○ | ● | ○ | ● | ● | ● | ● | ● | ● | ● | ● |
| Female | ● | ● | ● | ● | ● | ● | ● | ● | ● | ● | ● | ● |
| Race |  |  |  |  |  |  |  |  |  |  |  |  |
| *Ref: Black/African Am.* |  |  |  |  |  |  |  |  |  |  |  |  |
| White/Caucasian |  | × | × | × |  | ● | ● | ● |  | ● | ● | ● |
| Other |  | × | × | × |  | ● | ● | ● |  | ● | ● | ● |
| Not reported/declined |  | × | × | × |  | ● | ● | ● |  | × | × | × |
| Hispanic/Latino |  | × | × | × |  | × | × | × |  | ○ | ○ | ○ |
| Members per household |  |  |  |  |  |  |  |  |  |  |  |  |
| *Ref: Lives alone* |  |  |  |  |  |  |  |  |  |  |  |  |
| Two |  |  | × | × |  |  | ○ | ○ |  |  | × | × |
| Three to four |  |  | × | × |  |  | × | × |  |  | × | × |
| More than five |  |  | × | × |  |  | × | × |  |  | × | × |
| Migrant or seasonal work |  |  | × | × |  |  | ● | ● |  |  | × | × |
| Military discharge |  |  | × | × |  |  | × | × |  |  | × | × |
| Uninsured |  |  | × | × |  |  | × | × |  |  | ○ | ○ |
| Lacks high school education |  |  | × | × |  |  | × | × |  |  | × | × |
| Work situation |  |  |  |  |  |  |  |  |  |  |  |  |
| *Ref: Full-time* |  |  |  |  |  |  |  |  |  |  |  |  |
| Part-time |  |  | ○ | ○ |  |  | × | × |  |  | × | × |
| Unemp., seeking work |  |  | × | × |  |  | × | × |  |  | ● | ● |
| Unemp., not seeking work |  |  | ○ | ○ |  |  | ○ | ○ |  |  | ● | ○ |
| No housing |  |  |  | ● |  |  |  | × |  |  |  | × |
| Worried, losing housing |  |  |  | × |  |  |  | × |  |  |  | × |
| Lacks transportation |  |  |  | ○ |  |  |  | × |  |  |  | × |
| Low social interaction |  |  |  | × |  |  |  | × |  |  |  | × |
| High stress |  |  |  | ● |  |  |  | × |  |  |  | ○ |
| Feels unsafe at residence |  |  |  | × |  |  |  | × |  |  |  | ○ |
| Afraid of partner |  |  |  | × |  |  |  | × |  |  |  | × |
| Other material need |  |  |  |  |  |  |  |  |  |  |  |  |
| Food |  |  |  | × |  |  |  | × |  |  |  | ○ |
| Access to health care |  |  |  | × |  |  |  | ● |  |  |  | ○ |
| Utilities |  |  |  | × |  |  |  | × |  |  |  | × |
| Clothing |  |  |  | × |  |  |  | × |  |  |  | × |
| Child care |  |  |  | × |  |  |  | × |  |  |  | × |
| Phone |  |  |  | × |  |  |  | × |  |  |  | ○ |
| Other |  |  |  | × |  |  |  | × |  |  |  | × |
| Total Number of Var. | 2 | 2 | 4 | 7 | 2 | 5 | 8 | 9 | 2 | 5 | 8 | 13 |

**●** = included and statistically significant (P < 0.05), **○ =** included but not statistically significant (P < 0.157), **×** = removed from the model

**TABLE 2. Included Covariates for LASSO Nested Models^a^**

|  | Obesity | | | | Hypertension, Stage-1 | | | | ASCVD, Borderline | | | |
| --- | --- | --- | --- | --- | --- | --- | --- | --- | --- | --- | --- | --- |
|  | **M1** | **M2** | **M3** | **M4** | **M1** | **M2** | **M3** | **M4** | **M1** | **M2** | **M3** | **M4** |
| Age | ○ | ○ | ○ | ○ | ○ | ○ | ○ | ○ | ○ | ○ | ○ | ○ |
| Female | ○ | ○ | ○ | ○ | ○ | ○ | ○ | ○ | ○ | ○ | ○ | ○ |
| Race |  |  |  |  |  |  |  |  |  |  |  |  |
| *Ref: Black/African Am*. |  |  |  |  |  |  |  |  |  |  |  |  |
| White/Caucasian |  | × | × | × |  | ○ | ○ | ○ |  | ○ | ○ | ○ |
| Other |  | × | × | × |  | ○ | ○ | ○ |  | ○ | ○ | ○ |
| Not reported/declined |  | × | × | × |  | ○ | ○ | ○ |  | × | × | × |
| Hispanic/Latino |  | × | × | × |  | ○ | ○ | ○ |  | ○ | ○ | ○ |
| Members per household |  |  |  |  |  |  |  |  |  |  |  |  |
| *Ref: Lives alone* |  |  |  |  |  |  |  |  |  |  |  |  |
| Two |  |  | × | × |  |  | ○ | ○ |  |  | × | × |
| Three to four |  |  | × | × |  |  | × | × |  |  | × | × |
| More than five |  |  | × | × |  |  | × | × |  |  | × | × |
| Migrant or seasonal work |  |  | × | × |  |  | ○ | ○ |  |  | × | × |
| Military discharge |  |  | × | × |  |  | ○ | ○ |  |  | × | × |
| Uninsured |  |  | × | × |  |  | ○ | ○ |  |  | × | × |
| Lacks high school education |  |  | × | × |  |  | × | × |  |  | × | × |
| Work situation |  |  |  |  |  |  |  |  |  |  |  |  |
| *Ref: Full-time* |  |  |  |  |  |  |  |  |  |  |  |  |
| Part-time |  |  | × | × |  |  | × | × |  |  | × | × |
| Unemp., seeking work |  |  | × | × |  |  | × | × |  |  | × | × |
| Unemp., not seeking work |  |  | × | × |  |  | ○ | ○ |  |  | ○ | ○ |
| No housing |  |  |  | ○ |  |  |  | × |  |  |  | ○ |
| Worried, losing housing |  |  |  | × |  |  |  | × |  |  |  | × |
| Lacks transportation |  |  |  | ○ |  |  |  | × |  |  |  | × |
| Low social interaction |  |  |  | × |  |  |  | × |  |  |  | ○ |
| High stress |  |  |  | ○ |  |  |  | × |  |  |  | ○ |
| Feels unsafe at residence |  |  |  | × |  |  |  | × |  |  |  | × |
| Afraid of partner |  |  |  | × |  |  |  | × |  |  |  | × |
| Other material need |  |  |  |  |  |  |  |  |  |  |  |  |
| Food |  |  |  | × |  |  |  | × |  |  |  | ○ |
| Access to health care |  |  |  | × |  |  |  | ○ |  |  |  | ○ |
| Utilities |  |  |  | × |  |  |  | × |  |  |  | × |
| Clothing |  |  |  | × |  |  |  | × |  |  |  | × |
| Child care |  |  |  | × |  |  |  | × |  |  |  | ○ |
| Phone |  |  |  | × |  |  |  | × |  |  |  | ○ |
| Other |  |  |  | × |  |  |  | × |  |  |  | × |
| Total Number of Var. | 2 | 2 | 2 | 5 | 2 | 6 | 11 | 12 | 2 | 5 | 6 | 13 |

**○ =** included in the model, **×** = removed from the model

^a^ The LASSO algorithm does not involve or compute P-values for variable coefficients.
